# Inhibition of never in mitosis A (NIMA)-related kinase-4 reduces survivin expression and sensitizes cancer cells to TRAIL-induced cell death

**DOI:** 10.18632/oncotarget.11781

**Published:** 2016-09-01

**Authors:** So Jung Park, Doo Sin Jo, Se-Young Jo, Dong Woon Shin, Sangmi Shim, Yoon Kyung Jo, Ji Hyun Shin, Ye Jin Ha, Seong-Yun Jeong, Jung Jin Hwang, Young Sam Kim, Young-Ah Suh, Jong Wook Chang, Jin Cheon Kim, Dong-Hyung Cho

**Affiliations:** ^1^ Graduate School of East-West Medical Science, Kyung Hee University, Gyeonggi-do, 17104 S. Korea; ^2^ Asan Institute for Medical Research, University of Ulsan College of Medicine, Asan Medical Center, Seoul, 05505 S. Korea; ^3^ Department of Biomedical Sciences, College of Medicine, Seoul National University, Seoul, 03080 S. Korea; ^4^ Department of Internal Medicine, Yonsei University College of Medicine, Seoul, 03722 S. Korea; ^5^ Stem Cell & Regenerative Medicine Institute, Research Institute for Future Medicine, Samsung Medical Center, Seoul, 06351 S. Korea; ^6^ Department of Health Sciences and Technology, Samsung Advanced Institute for Health Sciences and Technology (SAIHST), Sungkyunkwan University, Seoul, 06351 S. Korea; ^7^ Department of Surgery, University of Ulsan College of Medicine, Asan Medical Center, Seoul, 05505 S. Korea

**Keywords:** NEK4, survivin, cancer, TRAIL

## Abstract

The tumor necrosis factor-related apoptosis inducing ligand (TRAIL) preferentially induces apoptosis in cancer cells. However, many tumors are resistant to TRAIL-induced apoptosis, and resistance mechanisms are not fully understood. To identify novel regulatory molecules of TRAIL resistance, we screened a siRNA library targeting the human kinome, and NEK4 (NIMA-related kinase-4) was identified. Knockdown of NEK4 sensitized TRAIL-resistant cancer cells and *in vivo* xenografts to cell death. In contrast, over expression of NEK4 suppressed TRAIL-induced cell death in TRAIL-sensitive cancer cells. In addition, loss of NEK4 resulted in decrease of the anti-apoptotic protein survivin, but an increase in apoptotic cell death. Interestingly, NEK4 was highly upregulated in tumor tissues derived from patients with lung cancer and colon cancer. These results suggest that inhibition of NEK4 sensitizes cancer cells to TRAIL-induced apoptosis by regulation of survivin expression.

## INTRODUCTION

During the past decades, the strategy for cancer treatment has changed from relatively nonspecific cytotoxic chemical agents to selective molecular target-based therapeutics [1]. The most significant obstacle to curative therapy for cancer is metastasis, which accounts for the majority of cancer-related mortality [2, 3]. Metastatic cancer cells obtain resistance to cell death through genetic and epigenetic changes, and impaired programmed cell death is one mechanism of therapeutic resistance in cancer cells [3, 4].

Apoptosis is a distinct process involving the genetically determined elimination of cells. As apoptosis is highly associated with many diseases including neurodegeneration and cancer, it is a tightly regulated process [5, 6]. Apoptosis is initiated through one of two pathways. Both the extrinsic and intrinsic pathways induce cell death by activating caspases, which are cysteine proteases that degrade essential proteins involved in homeostasis and survival [7, 8]. Not only apoptosis, but also autophagy and programmed necrosis are proposed to be programmed cell death mechanisms in cancer cells [9, 10]. The tumor necrosis factor (TNF)-related apoptosis-inducing ligand (TRAIL) is a member of the TNF family, which includes Fas ligand and TNF-α. TRAIL triggers apoptosis by binding to death receptor 4 (DR4) and DR5, eventually recruiting a death-inducing signaling complex, which activates the caspase cascade [5, 6]. Unlike Fas ligand and TNF-α, TRAIL preferentially targets cancer cells. Therefore, TRAIL has been considered a potential cancer therapeutic agent [11–13]. However, evading apoptosis is a hallmark of cancer [14]. Indeed, preclinical studies indicate that approximately half of non-small cell lung cancer (NSCLC) cell lines are intrinsically resistant to TRAIL-induced cell death, and several mechanisms underlying TRAIL-resistance in NSCLC have been suggested [15]. For example, dysfunction of TRAIL receptors and abnormal expression of anti-apoptotic proteins results in resistance to TRAIL in tumor cells [16]. In addition, intracellular factors acting downstream in the death receptor pathway are associated with TRAIL-resistance in NSCLC. Several mutations in apoptosis regulators, such as cellular FLICE-like inhibitory proteins are occasionally found in various types of tumor cells [17, 18]. These alterations are understandable because TRAIL is now characterized as part of the immune system response to primary tumors, suggesting that primary tumors have already evaded TRAIL. To overcome the resistance to TRAIL that develops in cancer cells, numerous studies have suggested that combination therapy with specific targeting agents significantly enhances the toxicity of TRAIL in cancer cells [15]. Nonetheless, the mechanism of overcoming TRAIL-resistance is not fully understood.

Kinases transduce cellular signaling and regulate complex cellular processes. Protein phosphorylation enhances or inhibits activity and modulates its ability to regulate other molecules [19]. In addition, various loss-of-function or gain-of-function mutations in kinases can cause cancer [20]. Thus, over the last decade, protein kinases have been the most important targets of the pharmaceutical industry for the treatment of cancer [19, 20]. To further understand the molecular mechanisms underlying TRAIL-resistance in NSCLC, we screened a siRNA library set consisting of 719 human protein kinase genes in TRAIL-resistant cancer cells. As a result, NIMA-related kinase-4 (NEK4) was identified as a novel candidate. We found that loss of NEK4 sensitized TRAIL-resistant lung cancer cells to cell death *in vitro* and *in vivo.* Suppressing NEK4 reduced the expression of survivin. Furthermore, NEK4 was upregulated in lung cancer and colon cancer tissues. These results suggest that downregulation of NEK4 sensitizes cancer cells to TRAIL-induced apoptosis by decreasing survivin.

## RESULTS

### Inhibition of NEK4 potentiates TRAIL-induced cell death in TRAIL-resistant cancer cells

**Figure 1:**
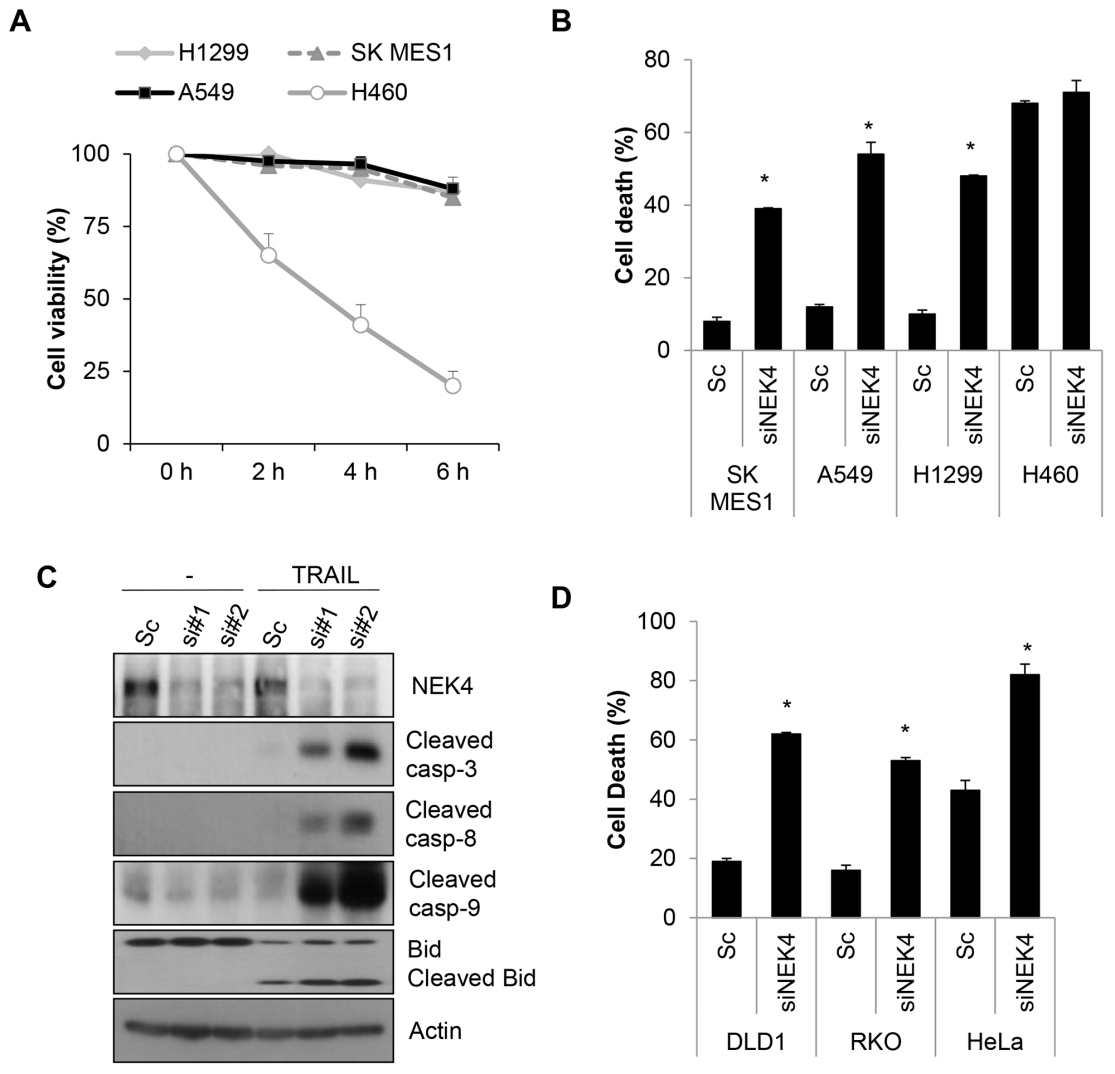
Downregulation of NEK4 sensitizes A549 cells to TRAIL-induced cell death **A.** Cell viability tests in various lung cancer cell lines. Several lung cancer cell lines (A549, H1299, H460, and SK-MES1 cells) were treated with TRAIL (20 ng/ml) for the indicated times, and cell viability was measured by a CCK-8 assay. **B.** SK-MES-1, A549, H1299, and H460 cells were transiently transfected with scrambled negative siRNA (Sc) or NEK4 siRNA (siNEK4), and the cells were treated 3 days later with TRAIL (20 ng/ml) for 4 h. Cell death was determined by Annexin V/PI staining. **C.** A549 cells transiently transfected with Sc or NEK4 siRNAs (si#1 and si#2) were further treated with TRAIL (20 ng/ml) for 4 h. The cells were harvested and subjected to Western blotting with the indicated antibodies. **D.** DLD1, RKO, and HeLa cells transiently transfected with Sc or siNEK4 were additionally treated with TRAIL (20 ng/ml) for 4h, and cell death was determined.

**Figure 2:**
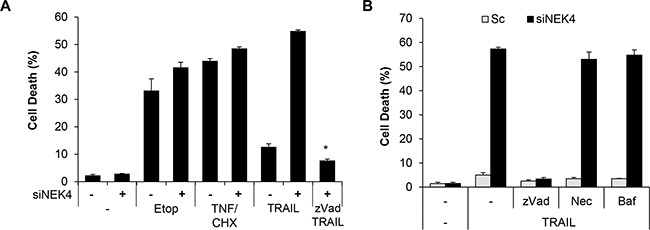
Downregulation of NEK4 induces apoptotic cell death in TRAIL-treated cells **A.** A549 cells transiently transfected with scrambled siRNA (−) or NEK4 siRNA (+) were further treated with 50 mM etoposide (Etop, 20 h), TNF and cyclohexamide (TNF/CHX, 30 ng/μl and 10 ng/μl, 4 h), and TRAIL (20 ng/ml, 4 h) in the presence or absence of zVAD (40 mM). Subsequently, cell death was determined at different time points. **B.** A549 cells transiently transfected with scrambled siRNA (▫) or NEK4 siRNA (▪) were treated with TRAIL (20 ng/ml) with or without cell death inhibitors (40 mM zVAD, 10 mM necrostatin-1 (Nec), or 100 nM bafilomycin (Baf)), and the number of dead cells was determined. Data are presented as the mean ± SEM (n = 3) and differences were considered significant at *p<0.05.

Although TRAIL preferentially kills cancer cells, a number of cancer cells are resistant to TRAIL-induced cell death. To investigate whether lung cancer cells are resistant to TRAIL-induced cell death, we examined the cytotoxic effect of TRAIL in lung cancer cells, including A549, H1299, H460, and SK-MES-1 cell lines. The cells were treated with TRAIL, and cell viability was determined. As a result, H460 cells were highly sensitive to TRAIL-induced cell death, whereas A549, H1299, and SK-MES-1 cells were strongly resistant to TRAIL-induced cell death (Figure 1A). To identify novel modulators of TRAIL sensitization, we screened a siRNA library comprising the human kinome (719 kinase genes). As kinases are drug targets and major regulators of cellular signaling, the kinome has been the focus of various studies on cancer. Based on screening results, we selected NEK4 as a novel regulator of TRAIL-mediated cell death. A549 cells were transiently transfected with NEK4 siRNA and exposed to TRAIL to verify the screening results. As shown in Figure 1B, knockdown of NEK4 induced cell death in TRAIL-resistant cancer cells (Figure 1B). In addition, activation of caspases-3, −8, and −9 and Bid cleavage were also dramatically enhanced in TRAIL-treated cells after depleting NEK4 (Figure 1C). Inhibition of NEK4 further potentiated TRAIL-induced cell death in colorectal cancer cells such as DLD1 and RKO cells, and HeLa cervical cancer cells (Figure 1D). To examine the effect of NEK4 knockdown on other cell death stimuli, A549 cells transiently depleted NEK4 were exposed to various cell death inducers, such as etoposide, which activates the intrinsic apoptotic pathway and TNF-α and cyclohexamide (TNF/CHX), which activate the extrinsic apoptotic pathway. Interestingly, loss of NEK4 did not affect cell death triggered by either the etoposide or the TNF/CHX treatments (Figure 2A). However, cell death induced by TRAIL in NEK4 knockdown cells was dramatically inhibited by the pan-caspase inhibitor zVAD (Figure 2A). These results indicate that NEK4 is only involved in regulating the TRAIL-mediated cell death pathway. Although TRAIL is a well-known inducer of apoptosis, previous studies have shown that the necrosis and autophagic cell death mechanisms are involved in TRAIL-induced cell death [21, 22]. Therefore, we further addressed which types of cell death occurred in TRAIL-treated cells by NEK4 knockdown. A549 cells with suppressed NEK4 expression were pretreated with cell death inhibitors, such as zVAD, necrostatin-1, and bafilomycin, and the cells were additionally incubated with TRAIL to induce cell death. As shown in Figure 2B, TRAIL-induced cell death in NEK4 knockdown cells was completely blocked by zVAD but not by the necrosis inhibitor, necrostatin-1 or the autophagy inhibitor, bafilomycin (Figure 2B).

It was recently reported that quercetin inhibits a panel of kinases including NEK4, NEK9, and ABL1 in cancer cells [23]. In addition, quercetin enhances TRAIL-induced cytotoxicity in various cancer cells [23, 24]. Therefore, we investigated the effect of quercetin on TRAIL-induced cell death in NSCLC cells. A549 cells were treated with quercetin in the presence or absence of TRAIL then, cell death and caspase activation were observed. The combined treatment of quercetin and TRAIL significantly increased death in A549 cells (Supplementary Figure S1A). Treating A549 cells with either TRAIL alone or quercetin alone minimally activated caspase-8 and caspase-3, whereas combined TRAIL and quercetin treatment strongly activated caspase-3 and -8 and resulted in Bid cleavage (Supplementary Figure S1B), suggesting that quercetin potentiates TRAIL-induced toxicity in A549 cells. Taken together, these results suggest that downregulation of NEK4 sensitizes TRAIL-exposed cancer cells to apoptosis.

### Overexpression of NEK4 suppresses TRAIL-mediated death

**Figure 3:**
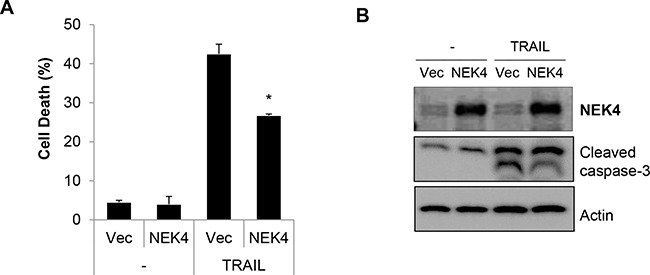
Overexpression of NEK4 suppresses TRAIL-induced cell death **A, B.** H460 cells were transfected with either a control vector (/Vec) or NEK4 (NEK4), and treated with TRAIL (20 ng/ml). After 2 h, cell death was determined (A). NEK4 and cleaved caspase-3 expression was confirmed by Western blotting (B). Data are presented as the mean ± SEM (n = 3) and were considered significant at *p < 0.05.

To further examine the effect of NEK in TRAIL-mediated cell death, we generated a stable cell line overexpressing NEK4 in H460 cells (H460/NEK4) and demonstrated a moderate response to TRAIL-induced cytotoxicity. The H460 and H460/NEK4 cells were treated with TRAIL, and cell death was investigated. Upregulation of NEK4 significantly reduced cell death and caspase-3 activation in TRAIL-treated cells (Figure 3A and 3B). In addition, we further confirmed the effect of ectopic expression of NEK4 on TRAIL-induced cell death in HeLa cells (Supplementary Figure S2), suggesting that overexpression of NEK4 suppresses TRAIL-mediated cell death.

### Survivin expression is decreased through inhibition of NEK4 in TRAIL-treated cells

**Figure 4:**
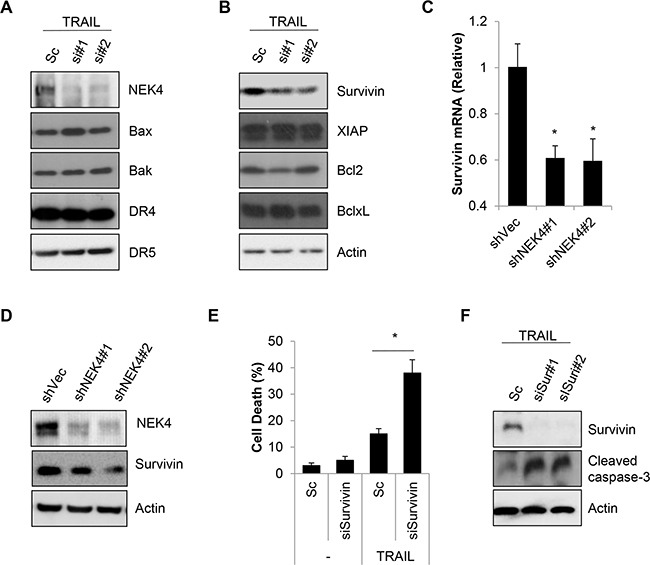
Down regulation of NEK4 decrease survivin expression **A, B.** A549 cells were transiently transfected with scrambled negative siRNA (Sc) or NEK4-specific siRNAs (si#1 and si#2). After 3 days, the cells were further treated with TRAIL (20 ng/ml) for 4 h. Subsequently, the cells were harvested for Western blotting with the indicated antibodies. **C.** A459 cells stably expressing control shRNA vector (A549/shVec) or NEK4 (A549/shNEK4) were treated with TRAIL (20 ng/ml) for 4 h. Then total mRNA was isolated from A549/shVec or A549/shNEK4 cells, and NEK4 mRNA was quantified by RT-PCR using NEK4 and GAPDH specific primers. **D.** Downregulation of the NEK4 protein in A549/shNEK4 cells was analyzed by Western blotting. **E, F.** A549 cells were transfected with scrambled siRNA (Sc) or siRNA for survivin (siSur#1, #2) and additionally exposed to TRAIL (20 ng/ml) for 4h. And cell death was measured (E). Reduced expression of survivin by siRNA and activation of caspase-3 were analyzed by Western blotting (F). * p < 0.05 vs. negative control. Data are presented as the mean ± SEM (n = 3) and differences were considered significant at *p < 0.05.

Resistance to TRAIL during cell death can be mediated by receptor sites as well as signaling pathways that are both upstream and downstream of the caspase cascade. To further address the mechanism responsible for TRAIL sensitization by NEK4 depletion, we investigated the expression of various targets of TRAIL-resistance, such as TRAIL receptors, anti-apoptotic and pro-apoptotic proteins. As shown in Figure 4A, expression levels of the TRAIL receptor and pro-apoptotic proteins, such as Bax and Bak, did not change by reducing NEK4 expression in TRAIL-treated cells (Figure 4A). Additionally, the expression level of some anti-apoptotic proteins, such as XIAP, Bcl-2, and Bcl-X_L_, was also unchanged. However, the anti-apoptotic protein survivin decreased significantly in response to inhibiting NEK4 in TRAIL-treated cells (Figure 4B). To confirm our previous results, we generated an A549 cell line with stable knockdown of NEK4, using a shRNA system (A549/shNEK4). We then investigated survivin expression in these cells. As shown in Figure 4C and 4D, survivin mRNA and protein levels were significantly decreased in A549/shNEK4 cells compared to those in A549 control cells (Figure 4C and 4D). We further investigated the effect of survivin down-regulation in TRAIL-induced cell death. Consistent with previous notion, knock down of survivin significantly increased TRAIL-induced cell death and caspase activation (Figure 4E and 4F), suggesting that survivin is associated with sensitization to TRAIL through inhibition of NEK4.

### Suppression of NEK4 potentiates the anti-cancer effect of TRAIL *in vivo*

**Figure 5:**
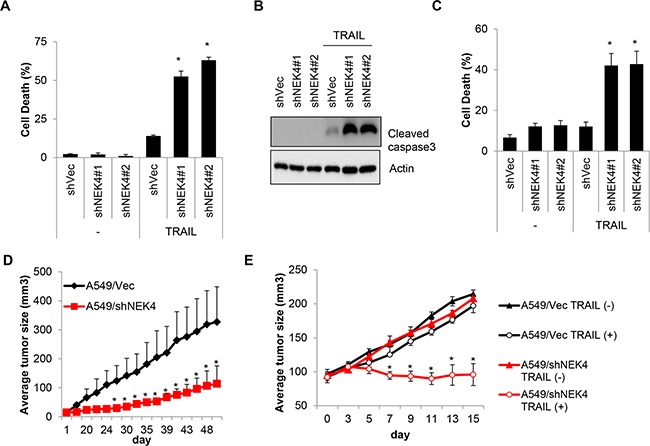
Loss of NEK4 increases TRAIL-induced cell death and inhibits tumorigenesis in a xenograft model **A.** A459 cells stably expressing control shRNA vector (A549/shVec) or NEK4 (A549/shNEK4) were treated with TRAIL (20 ng/ml) for 4 h, and the number of dead cells was determined. **B.** Reduced NEK4 expression in A549/shNEK4 cells was confirmed by Western blotting. **C.** A549/shVec and A549/shNEK4 cells were cultured in the GravityPLUS™ plate. After 7 days, the cells were treated with TRAIL (20 ng/ml) for 4 h and were collected. Cell death was determined by an MTT assay. **D.** Tumor growth after the implantation of A549/shVec and A549/shNEK4 cells was monitored for 50 days. **E.** When tumors grew to approximately 100 mm^3^ in size (5 weeks with A549/shVec and 7 weeks with A549/shNEK4 after inoculation), the mice (n=3) were treated intraperitoneally with PBS or TRAIL (400 mg). Tumor volumes were calculated measured everyday using the formula for an ellipsoid sphere: W1 × W 2 × W2/2, where W1 represents the largest tumor diameter and W2 the smallest tumor diameter.

To confirm the effect of NEK4 depletion on cell death, A549/Vec and A549/shNEK4 cells were treated with TRAIL, and cell death was measured. Consistently, the cytotoxic effect of TRAIL was increased significantly in A549/shNEK4 cells compared to that in A549/Vec cells (Figure 5A and 5B). Based on previous results, we additionally examined the effect of NEK4 in an *in vitro* 3D culture system. As shown in Figure 5C, down-regulation of NEK4 expression also notably enhanced cancer cell death after TRAIL treatment in 3D model systems (Figure 5C). Because the inhibition of NEK4 sensitize cells to TRAIL-mediated cell death *in vitro*, we further examined whether the depletion of NEK4 enhance the anti-tumor activity of TRAIL *in vivo*. Mouse xenograft models were established in CD-1 *nu/nu* mice using A459/Vec or A549/shNEK4 cells, and tumor growth and TRAIL responses were monitored. As shown in Figure 5D, A549 cells with NEK4 ablation formed tumors slowly, compared to wild type A549 cells (Figure 5D). We then investigated the effect of TRAIL on tumor growth. As tumor formation was different between A549/Vec and A549/shNEK4 xenografts, we injected TRAIL when tumors were reached approximately 100 mm^3^ in size (30–40 days after implantation). Interestingly, TRAIL alone did not efficiently inhibit growth of A549/Vec-xenografts in mice. By comparison, treatment of TRAIL in A549/shNEK4-xenograft mice was highly effective in inhibiting tumor growth (Figure 5E). Taken together, these results suggest that inhibition of NEK4 can enhance the anti-tumor activity of TRAIL in lung cancer.

### NEK4 is upregulated in human lung and colorectal cancer tissues

**Figure 6:**
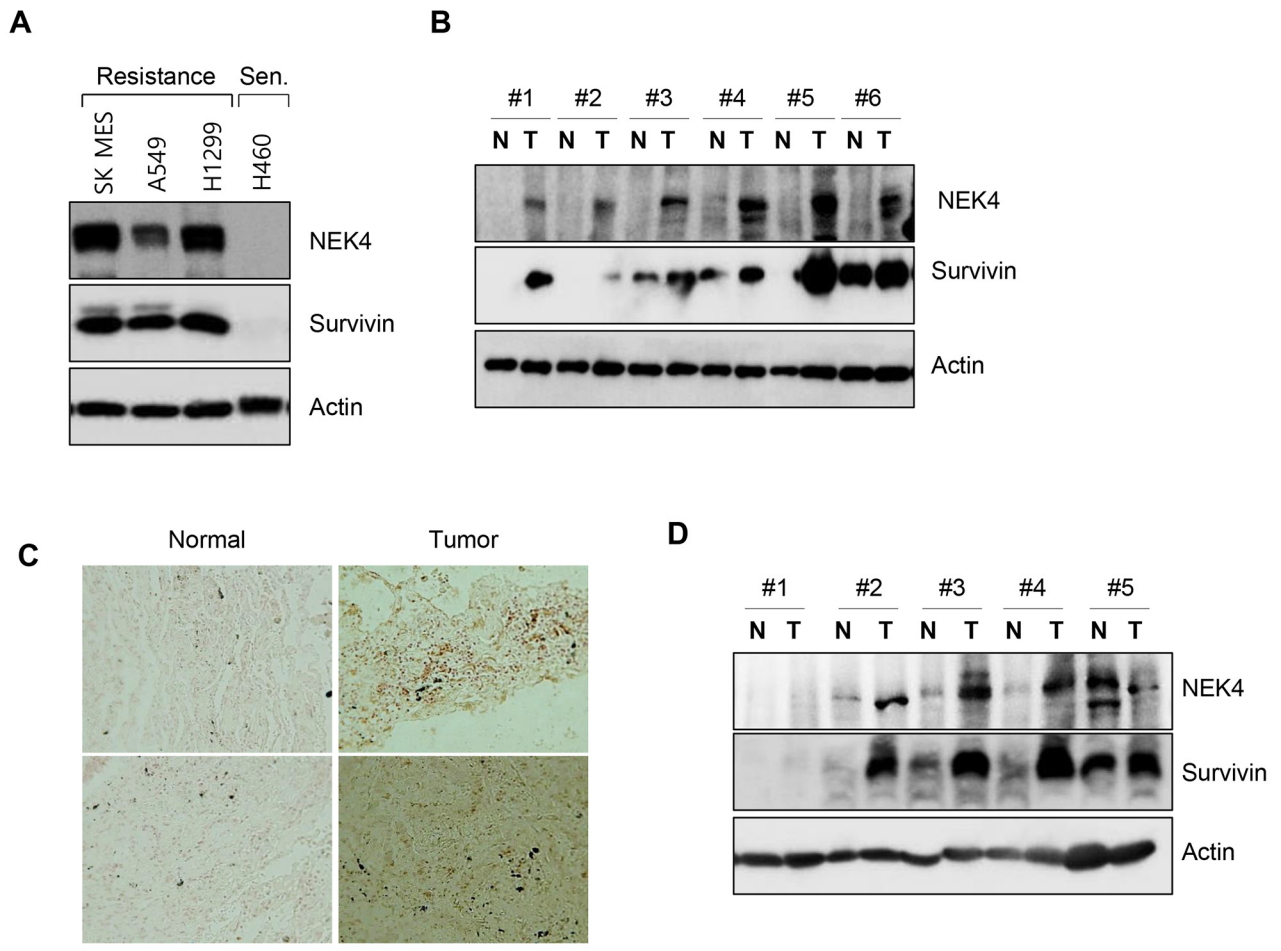
NEK4 expression is decreased in human lung and colon cancer tissues **A.** TRAIL-resistant lung cancer cell lines (SK-MES-1, A549, and H1299) and TRAIL-sensitive (Sen) lung cancer cells (H460) were cultured and harvested. NEK4 and survivin expression levels were assessed by Western Blot analysis. **B.** Expression of NEK4 and survivin was assessed by Western blotting in lung cancer tissues (T, tumor tissue; N, corresponding normal tissue). **C.** Representative photomicrographs of NEK4 immunohistochemical staining in lung cancer tissue. NEK4 in lung cancer tissue was examined by immunohistochemical analysis on a tissue microarray and its expression increased (right) in cancer tissues. **D.** Expression level of NEK4 and survivin in colon cancer was examined with Western blotting (T, tumor tissue; N, corresponding normal tissue).

As inhibition of NEK4 expression downregulated survivin expression, we next investigated survivin and NEK4 expression levels in several lung cancer cells and tissues. As shown in Figure 6A, NEK4 and survivin expression was significantly higher in TRAIL-resistant cells, including the SK-MES-1, A459, and H1299 cell lines (Figure 6A). However, NEK4 and survivin expression was virtually undetectable in TRAIL-sensitive H460 cells (Figure 6A). We next examined the expression of NEK4 and survivin in tumor tissues. In lung cancer tissue samples, both NEK4 and survivin expression were highly upregulated in tumors compared to those in adjacent normal tissues in six of eight cases (Figure 6B). Increased expression of NEK4 was additionally confirmed in lung cancer tissues of the tissue microarray (Figure 6C). As with lung cancer tissue, NEK4 expression was also elevated in colorectal tumor tissues, when compared to that in normal tissues (3/5 cases) (Figure 6D), suggesting that NEK4 is upregulated during tumorigenesis.

## DISCUSSION

Cancer is one of the most complex and dynamic diseases. In spite of rapid advances in the field of cancer research, the molecular mechanisms of tumorigenesis and acquired metastatic ability have not been fully elucidated; this has limited cancer treatment [25, 26]. Although apoptosis can inhibit tumor development and metastatic dissemination by inducing cell death in target cells, cancer cells frequently obtains the ability to overcome various stresses including loss of cell-cell contact, destruction by immune system, and oxygen and nutrients deficiency, all of which promote cell death [14, 26]. Thus, for successful tumor development, cancer cells have to obtain the ability to escape programmed cell death.

As TRAIL receptors are dominantly expressed in cancer cells, TRAIL is known to selectively mediate apoptosis in tumor, and not normal cells. However, according to pre-clinical studies performed in the past, tumor cells can develop resistance to TRAIL, similar to other anti-cancer agents. Resistance to anti-cancer drugs occurs through multiple complex processes, which involve various genetic, epigenetic, and signaling changes [27]. One possibility of drug resistance is the absence of a suitable biomarker [28, 29]. Thus, identification of the mechanisms behind this resistance might lead to the development of new strategies for cancer treatment. In this study, we investigated the underlying mechanism of TRAIL-resistance through siRNA screening. Interestingly, we discovered that downregulation of NEK sensitizes TRAIL-resistant cells to the overall effect of this ligand.

NEK is known as a NimA-related kinase. NimA, or Never in mitosis A is a kinase involved in the regulation of the cell cycle checkpoint. There are 11 reported NEK isozymes, which have N-terminal serine/threonine kinase domain [23]. NEK proteins are involved in cell cycle regulation, which suggests that their alteration contributes to tumor progression. Indeed, several NEK proteins such as NEK2, NEK6, and NEK8 are overexpressed or mutated in various types of cancer [30–33]. In this study we found that knock down of NEK4 accelerated the sensitization of cancer cells to TRAIL both *in vitro* and *in vivo*, whereas ectopic expression of NEK4 blocked cell death (Figure 1, 3, and 5). Quercetin is a flavonoid and has diverse biological effects. Actually it is well defied that quercetin can act on the chemo-sensitization and radio-sensitization in various cancer cells [34, 35]. Quercetin inhibits protein kinases involved in deregulated cell growth in cancer cells, and NEK4 is a known target of quercetin [23]. Accordingly, we also found that treatment with Quercetin enhanced the sensitizing effect of TRAIL in TRAIL-resistant lung cancer cells (Supplementary Figure S2). However, our results suggested that kinase activity of NEK4 may not be involved in TRAIL-sensitization. Unlike the effect of knockdown, overexpression of a kinase dead mutant of NEK4 (K35A) did not efficiently alter the cell death response after treatment (Supplementary Figure S3). Thus, it is necessary to further explore the precise mechanism through which NEK4 regulates TRAIL sensitization.

To understand the molecular mechanism behind the ability of NEK4 to mediate sensitivity to TRAIL in cancer cells, the expression of TRAIL receptors, pro-apoptotic, and anti-apoptotic proteins were analyzed after NEK4 knockdown and treatment with TRAIL. However, the expression of most of these proteins did not significantly change except that of survivin (Figure 4). Survivin, an anti-apoptotic protein, is highly expressed in most cancers and is associated with chemotherapy resistance, increased tumor recurrence, and shorter patient survival, making anti-survivin therapy an attractive cancer treatment strategy for use with a chemotherapeutic regimen [36–38]. In addition, survivin is a tumor-specific molecule that promotes tumor-associated angiogenesis [39]. Here, we found that the expression of survivin was elevated in NEK4 knockdown cells, and that loss of survivin also sensitized A549 cells to TRAIL-induced cell death. Furthermore, expression analysis revealed that both NEK4 and survivin levels were elevated in lung cancer cell lines and patient tissues (Figure 6). TRAIL-resistant cells such as SK- MES-1, A549, and H1299 cells showed high expression of both NEK4 and survivin, whereas the extream opposite was observed in H460 cells which are TRAIL-sensitive cells (Figure 6). In addition, expression analysis in cancer tissues showed that NEK4 and survivin were elevated in tumor tissues compared to tumor-adjacent normal tissues (six of eight lung cancer patients and three of five colon cancer patients) (Figure 6). However, in this study, we analyzed the expressions of these proteins in only a small set of cancer tissues. Thus, further studies are needed to identify upstream promoters that regulate NEK4 and survivin expression. Moreover, investigation is also required to assess the relationship between NEK4 and survivin expression in various types of cancers, using a large number of samples.

In conclusion, the results from the current study suggest the potential of NEK4 as a candidate biomarker to screen patients with TRAIL resistance.

## MATERIALS AND METHODS

### Reagents

TRAIL, TNF-α, and zVAD were purchased from R&D Systems (Minneapolis, MN, USA). Etoposide, cyclohexamide, necrostatin-1, bafilomycin A1, and quercetin were obtained from Sigma-Aldrich (St. Louis, MO, USA). The human NEK4 gene was purchased from Imagenes (Santa Fe Springs, CA, USA) and was inserted into HA-tagged pcDNA3 (pcNDA/HA-NEK4) (Invitrogen, Carlsbad, CA, USA). The pSUPER-shRNA plasmid was purchased from OligoEngine (Seattle, WA, USA), and the NEK4 target sequence (sense sequence: 5′-GAAGAAAGGCCGTCTGTGA-3′) was cloned into the BglII/HindIII sites of pSUPER-shRNA (pSUPER/shNEK4) according to the system protocol. From the sequence alignment analysis of NEK family protein, NEK4 mutant lacking kinase activity (NEK4 K35A, kinase domain) was generated using A Quickchange Site-Directed Mutagenesis kit (Stratagene, La Jolla, CA) [40].

### Cell culture and establishment of stable transfected cells

A549, H1299, SK-MES-1, H460, and HeLa cells were obtained from the American Type Culture Collection (Manassas, VA, USA) and maintained in Dulbecco's modified Eagle's medium (Invitrogen) with 10% fetal bovine serum (Hyclone, Logan, UT, USA), 100 units/ml penicillin, and 100 μg/ml streptomycin at 37°C in 5% CO_2_. HeLa cells were transfected with pcDNA/HA-NEK4 using Lipofectamine reagent, and stable transfectants were selected by incubation with 1 mg/ml G418 (Invitrogen) for 10 days, with medium changed every 2 days. To generate NEK4 stable knockdown cells, A549 cells were transfected with pSUPER/shNEK4, and stable transfectants (A549/shVec and A549/shNEK4) were selected by incubation with 1 mg/ml G418. Stable cell lines were confirmed by Western blotting using HA or NEK4 antibody. For the 3D *in vitro* culture model, we employed a Hanging Drop System, ‘GravityPLUS™ Kit’ (InSphero, Brunswick, ME, USA). According to the manufacture's protocol, A549/shVec and A549/shNEK4 cells were seeded in the GravityPLUS™ plate. After 7 days for assessing microtissue formation, the cells were treated with TRAIL and collected to analyse cell death.

### siRNA library screening

We used an arrayed library of 719 siRNA pools covering the vast majority of the human kinase siRNA for primary screening (Dharmacon, Thermo-Fisher Scientific, Rockford, IL, USA). Each pool consisted of four oligonucleotides targeting a different region of the same gene. Each assay plate included the following controls: non-targeting siRNA and siRNA for FADD (transfection efficiency control) siRNA. siRNAs (50 pmol) were transiently transfected into 2,000 cells/well in 96-well plates using Lipofectamine 2000 in Opti-MEM. After 2 h incubation, the cells were incubated for 48 h under standard culture conditions and treated with 20 ng/ml TRAIL for 4 h. Then, cell viability was determined. The NEK4 siRNAs (sequences:#1, 5′-GAACAAACAUCAUCAAAG U-3′; #2, 5′-GGAGGUAUAUGCAGUAUUU-3′), and survivin siRNAs (#1, 5′- GAAGCAGUUUGAAGAAUUA-3′; #2, 5′- AGAAGCAGUUUGAAGAAUU-3) were purchased from Bioneer (Daejeon, Korea).

### Western blotting

Lysates were prepared with 2× Laemmli sample buffer (62.5 mM Tris-HCl, pH 6.8, 25% glycerol, 2% SDS, 5% β-mercaptoethanol, and 0.01% bromophenol blue) (Bio-Rad, Hercules, CA, USA). Protein (approximately 50 μg) was quantified using Bradford's solution (Bio-Rad) according to the manufacturer's instruction. The samples were separated by SDS-polyacrylamide gel electrophoresis and transferred to PVDF membranes (BioRad). After blocking with 4% skim milk in TBST (25 mM Tris, 3 mM 140 mM NaCl, and 0.05% Tween20), the membranes were incubated overnight with specific primary antibodies. [anti-actin (MAB1501) antibody; Millipore, Temecula, CA, USA; anti-Bcl-xl (#2764), anti-Bcl2 (#2870), anti-Bax (#2772), anti-Bik (#4592), anti-Bak (#6947), anti-survivin (#2803), anti-cleaved caspase-3 (#9661), and anti-Mcl-1(#5453) antibodies; Cell Signaling Technology, Danvers, MA, USA; anti-DR4 (ab13890) and anti-DR5 (ab47179) antibodies; Abcam, Cambridge, MA, USA; and anti-XIAP (61062) antibody; BD Biosciences, San Jose, CA, USA. The caspase antibody sampler kit was obtained from Imagenex (San Diego, CA, USA). Anti-NEK4 antibody was purchased from Bethyl Laboratories (Montgomery, TX, USA). The membranes were incubated with HRP-conjugated secondary antibodies (Pierce, Rockford, IL, USA).

### Cell viability and cell death assays

Cells were seeded in a 96-well plate, and cell viability was measured with a plate reader (absorbance at 450 nm) after incubation with the Cell counting kit-8 solution reagent (10 μl) (Dojindo Laboratories, Kumamoto, Japan) for 2 h. Cell death was assessed by staining with propidium iodide (PI) solution (50 μg/ml) and Annexin V (BD Pharmingen, San Diego, CA, USA) for 30 min. After washing with PBS, the cells were analyzed on a flow cytometer using CellQuest software (Becton Dickinson, Franklin Lakes, NJ, USA).

### Xenograft model system

Athymic male nude mice (CD-1 *nu*/*nu* from the Jungang Inc., Seoul, Korea) were used for *in vivo* tumor growth studies. All animal experiments were performed following approved institutional experimental animal care and use protocols. A549/shVec or A549/shEK4 cells were resuspended at 5 × 10^6^ per 200 μl in phosphate-buffered saline (PBS) and injected into the legs of 4-week-old mice. When tumors were approximately 100 mm^3^ in size (30–40 days), the animals were randomly allocated into two groups. Following randomization, mice were treated with vehicle or TRAIL (total 400 mg) was treated as four times in every two or three days by intraperitoneal injection. Tumor size was measured with digital calipers. Tumor measurements were converted to tumor volume using the following formula: W1 × W2 × W2/2 = x mm^3^ (where W1 and W2 represent the largest and the smallest tumor diameters, respectively). Mice were monitored up to 1 week after treatment.

### Reverse transcription-polymerase chain reaction (PCR) analysis

Total RNA was isolated from Chang cells using a PureLink RNA Mini Kit (Invitrogen). Total RNA sample (5 μg) was reverse transcribed with SuperScript First-Strand Synthesis System for RT-PCR (Invitrogen) followed by PCR amplification. The oligonucleotide primer sequences were as follows: 5′-CGAGTGTTAG AGAACCAC-3′ (sense) 5′-CTTATCAGTT CTGCCAGC-3′ (antisense) for NEK4 and 5′-AGGGCTGCTTTTAACTCTGGT-3′ (sense) 5′-CCCCACTTGATTTTGGAGGGGA-3′ (antisense) for GAPDH. PCR was carried out in a 2720 thermal cycler (Applied Biosystems, Foster City, CA, USA).

### Lung and colon tissues

Lung and colorectal cancer tissue samples were chosen randomly from patients treated at Severance Hospital, Yonsei Medical University (Seoul, Korea) and Asan Medical Center (Seoul, Korea). Primary adenocarcinoma tissue and adjacent normal tissue (at least 10 cm from the tumor) were obtained during surgery. All patients provided written informed consent, and the study protocol was approved by the Institutional Review Board for Human Genetic and Genomic Research in accordance with the Declaration of Helsinki.

### Tissue array and immunohistochemistry

A lung cancer tissue array paired with normal tissues was purchased from Abcam (Cambridge, MA, USA). The tissue array consisted of 16 cases samples in which one normal tissue core was paired with two tumor tissue cores from each patient and each sample was accompanied by grading and TNM classification staging data. Paraffin-embedded tissue cores were subjected to immunostaining according to the standard manufacturer's protocol based on the labeled streptavidin-biotin method with a DAKO LSAB^®^ kit (Dako, Carpentaria, CA, USA) according to the manufacture's protocol.

### Statistical analysis

All results are expressed as mean ± standard error. The statistical analysis was performed using multiple comparisons tests and SPSS17.0 software (SPSS Inc., Chicago, IL, USA). A *P-*value < 0.05 was considered significant.

## SUPPLEMENTARY MATERIALS FIGURES


