# Supplementary material for: Inhibition of never in mitosis A (NIMA)-related kinase-4 reduces survivin expression and sensitizes cancer cells to TRAIL-induced cell death
Source: Oncotarget. 2016 Sep 1;7(40):65957–67. doi: 10.18632/oncotarget.11781 (PMC5323206; doi:10.18632/oncotarget.11781)
Supplement: Supplementary file 1 [file oncotarget-07-65957-s001.pdf]

Inhibition of never in mitosis A (NIMA)-related kinase-4 reduces survivin expression and sensitizes cancer cells to TRAIL-induced cell death

SUPPLEMENTARY FIGURES

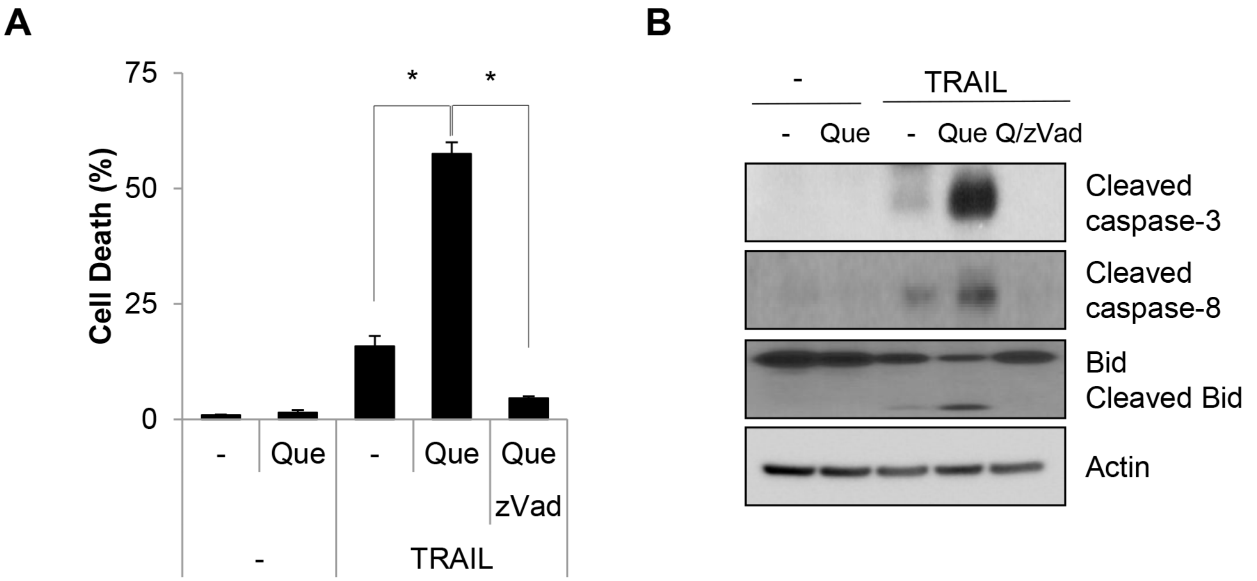

Supplementary Figure S1: Quercetin treatment increases TRAIL-mediated death in A549 cells. A, B. A549 cells were treated with quercetin (Que, 50 mM) alone or Que and zVAD (40 mM) and were additionally exposed to TRAIL (20 ng/ml) for 4 h. Cell death was determined by FACS analysis and Annexin-V and PI staining (A). The cells were further analyzed by Western blotting with the indicated antibodies (B).

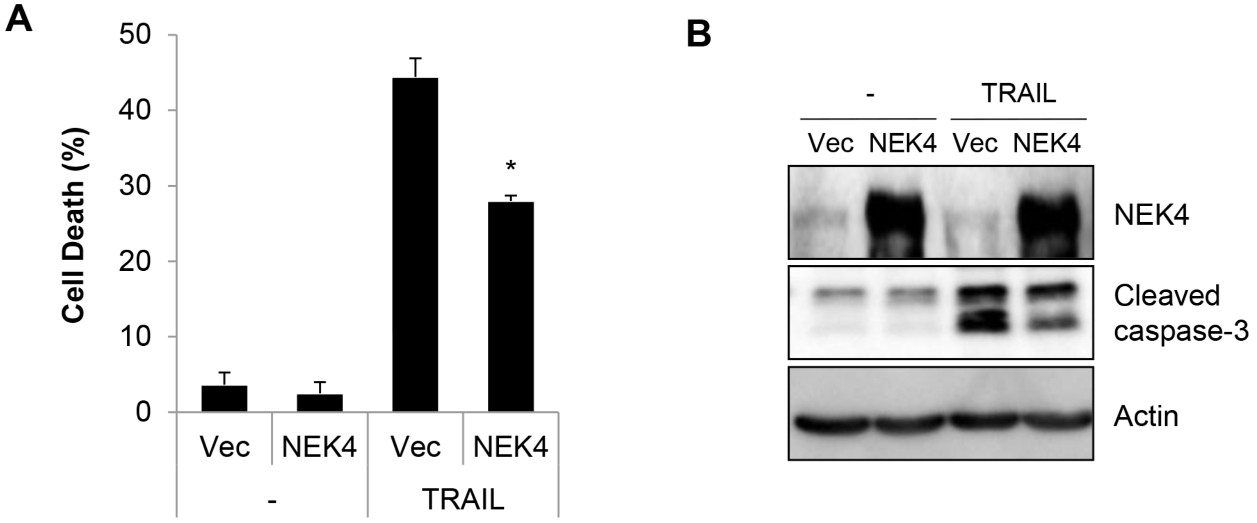

Supplementary Figure S2: Overexpression of NEK4 suppresses TRAIL-induced cell death. A. HeLa cells stably overexpressing either a control vector (HeLa/Vec) or NEK4 (HeLa/NEK4) were generated, and treated with TRAIL (20 ng/ml). After 6 h, cell death was determined by the FACS assay. **B.** HeLa/Vec and HeLa/NEK4 cells were incubated with TRAIL (20 ng/ml) for 6 h, and NEK4 and cleaved caspase-3 expression was confirmed by Western blotting. Data are presented as mean ± SEM ($n = 3$) and were considered significant at $*p < 0.05$.

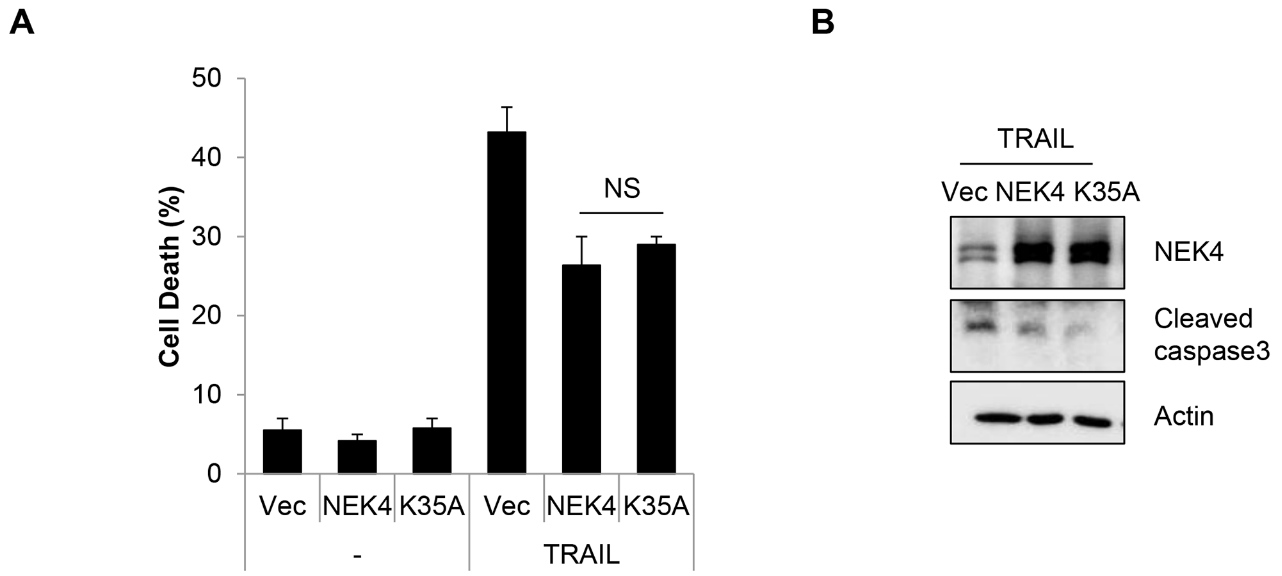

Supplementary Figure S3: A kinase dead NEK4 mutant does not alter TRAIL-mediated cell death. A, B. HeLa cells transiently transfected with control a vector (Vec), wild type NEK4 (NEK4), or NEK4 kinase dead mutant (K35A) were treated with TRAIL (20 ng/ml). After 4h treatment, cell death ratio was determined (A). The expression level of NEK4 and cleaved caspase-3 was analyzed by Western blotting (B).
